# Multi-Thoracolumbar Variations and *NR6A1* Gene Polymorphisms Potentially Associated with Body Size and Carcass Traits of Dezhou Donkey

**DOI:** 10.3390/ani12111349

**Published:** 2022-05-25

**Authors:** Ziwen Liu, Qican Gao, Tianqi Wang, Wenqiong Chai, Yandong Zhan, Faheem Akhtar, Zhenwei Zhang, Yuhua Li, Xiaoyuan Shi, Changfa Wang

**Affiliations:** 1 Liao Cheng Reaserch Inisitute of Donkey High-Efficiency Breeding, Liaocheng University, Liaocheng 252059, China; liuziwen345@163.com (Z.L.); qitianwang9797@163.com (T.W.); chaiwenqiong@lcu.edu.cn (W.C.); zhanyandong@lcu.edu.cn (Y.Z.); faheem_dear@hotmail.com (F.A.); qinyibushuo@163.com (Z.Z.); liyuhua1@lcu.edu.cn (Y.L.); xiaoyuans2021@163.com (X.S.); 2 Key Laboratory of Forage and Endemic Crop Biotechnology, Ministry of Education, School of Life Sciences, Inner Mongolia University, Hohhot 010070, China; gaoqican1993@163.com

**Keywords:** *NR6A1*, Dezhou donkey, thoracolumbar vertebrae variations, body size, SNV

## Abstract

**Simple Summary:**

The number of thoracic vertebrae (TN) and lumbar vertebrae (LN) in Dezhou donkey population is different, which leads to the difference of meat production and skin yield, and it is regulated by a few genes. Nuclear receptor subfamily 6 group A member 1 (*NR6A1*) was found to be related to livestock vertebra development, but it is not reported in donkeys yet. In this study, seven single nucleotide variants (SNVs) were detected in the *NR6A1* gene, and polymorphism information content (PIC) was moderate to high in the population. Then we analyzed the relationship between these SNVs and body size trait, carcass traits and the number of thoracolumbar vertebrae (TLN). We found that locus were associated with different traits, and the mutation effect was not completely consistent. The results suggested that these genetic variations in the *NR6A1* gene may play an important role in regulating the development of thoracolumbar vertebrae of Dezhou donkey. This paper provides important preliminary work for the study of multi thoracolumbar vertebrae in Dezhou donkeys.

**Abstract:**

The number of thoracolumbar vertebrae is a quantitative trait positively correlated with the economic traits of livestock. More thoracolumbar vertebrae individuals could genetically be used to improve the livestock population, as more thoracolumbar vertebrae means a longer carcass, which could bring more meat production. Nuclear receptor subfamily 6 group A member 1 (*NR6A1*) is considered a strong candidate gene for effecting the number of vertebrae in livestock. The purposes of this study are as follows: (a) Analyzing the effect of TLN variation on body size and carcass traits of Dezhou donkey; (b) Studying the distribution of seven single nucleotide variants (SNVs) in *NR6A1* gene of Dezhou donkey; (c) Exploring the relationship between latent SNVs and TLN, the body size and carcass traits. We examined the thoracic and lumbar vertebrae number and seven SNVs in *NR6A1* gene of 455 Dezhou donkeys, and analyzed the relationships between them. Five types of thoracolumbar combinations (T17L5 (individual with 17 thoracic and five lumbar vertebrae) 2.4%, T18L5 75.8%, T19L5 1.1%, T17L6 11.9%, and T18L6 8.8%) of Dezhou donkeys were detected in this study. For one thoracolumbar vertebra added, the body length of Dezhou donkey increases by 3 cm and the carcass weight increases by 6 kg. Seven SNVs (g.18093100G > T, g.18094587G > T, g.18106043G > T, g.18108764G > T, g.18110615T > G, g.18112000C > T and g.18114954T > G) of the *NR6A1* gene were found to have a significant association with the TLN, body size and carcass traits of Dezhou donkey (*p* < 0.05), respectively. For instance, g.18114954C > T is significantly associated with lumber vertebrae number, the total number of thoracolumbar, and carcass weight, and individuals with TT genotype had significantly larger value than CC genotype (*p* < 0.05). Using these 7SNVs, 16 different haplotypes were estimated. Compared to Hap3Hap3, individuals homozygous for Hap2Hap2 showed significantly longer length in one thoracic spine (STL), the total thoracic vertebrae and one thoracolumbar spine. Our study will not only extend the understanding of genetic variation in the *NR6A1* gene of Dezhou donkey, but also provide useful information for marker assisted selection in donkey breeding program.

## 1. Introduction

Domestic donkeys are one of the most common species in agricultural production and transportation especially in the small-scale peasant economic model [1]. They were used to pull or bear heavy cargos in agricultural activities. As agricultural machines become the major source of power in the current society, donkeys have been utilized for the purposes of food production, such as meat, milk and skin. There are 24 local donkey breeds in China, and the Dezhou donkey is one of the most typical large donkey breeds (body height greater than 130 cm), which is also an important donkey genetic resource in China. Population genomics analysis indicate that donkeys were domesticated in Africa, which is consistent with archeological research results [2,3]. In 2020, the stock of donkey in China is 2.32 million. In Shandong province of China, the output value of the whole donkey industry in 2020 was about 16 billion US dollars. 

In mammals, the vertebrate spinal column consists of five parts: the cervical, thoracic, lumber, sacral, and caudal [4,5]. The number of cervical vertebrae are nearly constant at seven [6]; increase in the sacral and caudal vertebrae number could not effectively improve the meat production and skin yield. However, one more thoracic vertebra means one more spine in vivo axis, and the increase in thoracic or lumbar vertebrae cannot only increase the body length, but also provide greater accommodation space for organs. Body length is an important factor affecting livestock slaughter performance [7], as a longer carcass means longer loins and more meat production, a larger body size means a larger body surface area and more skin yield. A variation in vertebrae number is found in a few domestic animals such as pigs [8,9,10], sheep [11,12,13], and cattle [14]. In European commercial pig breeds, a 14% variation in body length can be attributed to vertebrae number variation, for each additional vertebrae, there was an increase in length of about 15 mm [15]. In Asia Kazakh sheep, the carcass length of sheep possessing 20 thoracolumbar vertebrae increased 2.22–2.93 cm compared with that of sheep possessing 19 thoracolumbar vertebrae [13]. Few studies reported vertebrae number variation in equine animals. Stecher reported variation in the lumbo-sacral spine of the Przevalski and domestic horse [16]. Sisson reported variation in the number of lumbar vertebrae in the Arabian horse, mule and donkey (Sisson et al., 1975). Jamdar and Ema reported variation in thoracic and lumbar vertebrae number in 12 donkeys, the range of thoracic vertebrae number was 17~19 and the rage of lumbar vertebrae number was 5~6 [17]. However, the relationship between carcass weight and vertebrae number variation in Chinese donkeys remained unexplored.

Multi-vertebrae trait is complex and regulated by a lot of genes and signal pathways. Several genes have been identified to regulate development of the vertebrae in animals. NR6A1 is an important candidate gene affecting the development of the thoracolumbar vertebrae. There have been many studies on *NR6A1* and vertebrae number in livestock. Mikawa et al. mapped the genetic causes of different vertebrae between commercial pigs (Large White, Duroc) and most domestic pigs to the *NR6A1* gene [18,19,20,21]. Yang et al. detected allele frequencies of the *NR6A1* causative mutation (c.575T > C) in 519 pigs from three Western and seven Chinese breeds, and found allele T for increased vertebral number was fixed in Western breeds, but scare in most of the Chinese native breeds [21]. The association analysis revealed that chr1:299291323C > T, located in the second intron of *NR6A1*, were significantly associated with the number of vertebrae in the Tongcheng × Large White crossbreed population [22]. These findings suggest that the *NR6A1* gene is considered a strong candidate for affecting the number of vertebrae in donkeys. Through the previous study of our research group, several SNVs were detected in NR6A1 [2]. Therefore, the current study aimed to detect vertebrae number and seven mutation loci in the *NR6A1* gene of Dezhou donkeys and analyze their association with body size and carcass traits, as well as the thoracolumbar vertebrae, which will provide some useful information on the study of the mechanism of thoracolumbar development in donkeys.

## 2. Materials and Methods

### 2.1. Moral Statement 

The research protocols and animals used in this study were approved by the Animal Welfare and Ethics Committee of Institute of Animal Sciences, Liaocheng University (No. LC2019-1).

### 2.2. Animal and Data Collection

Blood samples were collected from Dong-e donkey factory in Dezhou City, Shandong province, China, including 455 unrelated samples: 20–24 months old male donkeys, which were fattening for meat production, with unknown pedigree information. During this study, the feed of animals consisted of grass and hay ad libitum, and water. Animals were slaughtered after 12 h of dietary prohibition (drinking water is not restricted). A 10 mL blood sample was collected into an EDTA anticoagulated blood collection tube from each Dezhou donkey by the jugular vein, and stored at −80 °C [23]. The body sizes of 455 Dezhou jackasses were recorded by ourselves for subsequent association analysis, including: body height (BH), body length (BL), carcass weight (CW), chest circumference (CHC), skin weight (SW), lumber vertebrae number (LN), lumber vertebra length (LL), thoracic vertebra number (TN), thoracic vertebra length (TL), and the total number of thoracic vertebra and lumber vertebra (TLN). For all samples, the body size measurements were taken by Zhang’s method [24]. The procedures of collecting carcass traits data were as follows, immediately after slaughter, hot carcass weight and the information of thoracolumbar vertebrae were measured. The head, viscus, skin, hooves, penises, testicles and tail were removed, and the carcass were weighted to obtain the carcass weight. The counting of TLN, TL, and LL of the 455 Dezhou donkeys was carried out at the abattoir in the cold-storage room immediately after slaughter on the left half of the carcass.

### 2.3. DNA Extraction

The 455 genomic DNA samples of Dezhou donkeys were extracted from whole blood with M5 FlexGen Blood DNA kit (TIANGEN, Beijing, China). Quality and purity of the extracted DNA was assessed using agarose gel electrophoresis (1%) and calculating the OD_260_/OD_280_ nm ratio determined with the Nanodrop (ND2000, NanoDrop, USA). After that, we diluted the DNA to a common centration 50 ng/μL and stored it at −20 °C.

### 2.4. PCR Amplification and Sequencing

Our research group resequenced the genome of 126 domestic donkeys, analyzed these data with reference genome (GenBank accession number: NC_052186.1) and generated a collection of 17.28 million SNVs and 1.5 million Indels [2], a total of 34 SNVs and 10 Indels were located on the *NR6A1*. Primer Premier 5.0 software was used to design 13 pairs of specific primers to amplify the total region on *NR6A1* gene (Table S1). To detect the polymorphisms, we used the above primers to design PCR amplification reactions. Reaction system is 25 μL, including 12.5 μL 2× Taq Master Mix (TIANGEN, without-dye, MF002, Beijing, China), 9.5 μL ddH_2_O, 50 ng/μL DNA template 1 μL, 10 μmol/L upstream primer (Sangon Biotech, Shanghai, China) 1 μL, 10 μmol/L downstream primer (Sangon Biotech, Shanghai, China) 1 μL. The PCR protocol was as follows: 95 °C for 5 min, 30 cycles of denaturing at 95 °C for 30 s, annealing at Tm (Table 1) for 30 s, and extension at 72 °C for 30 s, with a final extension at 72 °C for 10 min. After completing the whole reaction, PCR products were verified by electrophoresis in 1% agarose gel stained with ethidium bromide. The PCR product size was compared with the 2000 bp DNA ladder (Mei5bio, MF025, Beijing, China). Finally, the products were sent to the sequencing company (Beijing Genomics institution, Beijing, China) for sequencing, and results were analyzed using DNAstar software (Version 12.1, DNASTAR, Inc, USA). A total of 455 individuals were genotyped and used for the following correlation analysis. In this study, we showed 7 SNVs, which had a genotype frequency of one loci greater than 5% for each genotype and were associated with TLN, body size or carcass traits. The information of amplicon used for PCR of these seven SNVs are shown in Table 1.

### 2.5. Statistical Analysis

Correlation between the number variation in thoracolumbar vertebrae and body size and carcass traits using the Pearson Correlation coefficient analysis program of SPSS 22.0 software (Statistical Product and Service Solutions, Version 22.0 Edition, IBM, Armonk, NY, USA). The genotype frequency and allelic frequency of polymorphic loci were calculated and then the Chi-square Test was used to verify whether the allelic frequency of the sample population conforms to the Hardy–Weinberg equilibrium (HWE) by the Popgene software (Version 1.31, Molecular Biology and Biotechnology Centre, University of Alberta and Center for International Forestry Research, Canada). At the same time, the population genetic parameters were estimated, including observed heterozygosity (Obs-He), predicted heterozygosity (Pred-He), effective number of alleles (Ne) [25] and polymorphism information content (PIC) [26]. Haploview software (Version 4.2, Daly lab at the Broad Institute Cambridge, USA) was used to calculate the linkage disequilibrium between pairs of seven loci and construct haplotypes [27]. SPSS 22.0 software (IBM Statistics, Armonk, NY, USA), was also used to analyze the association between genotypes and body size traits. The linear analysis model can be written as y_ij_ = μ + a_i_ + e_ij_, where Y is the observation of the phenotypic traits, μ represents average deviation, a represents the fixed factor genotype, and b is the random error (due to the age, sex and farm of all samples were the same, these three factors were not included in the GLM model). Multiple comparisons of the associations were based on Bonferroni-corrected *p*-Values.

## 3. Results

### 3.1. Variation in Vertebral Number of Dezhou Donkey

The variation in vertebral number among 455 male Dezhou donkeys are shown in Table 2. The thoracic, lumber vertebral numbers ranged from 17–19 and 5–6, and dominated by 18 and five. The individuals with five lumbar vertebrae accounted for 79.3%, six lumbar vertebrae accounted for 20.7%, 17 thoracic vertebrae accounted for 14.3%, 18 thoracic vertebrae accounted for 84.6%, and 19 thoracic vertebrae accounted for 1.1%. The thoracic-lumber vertebral number have three types: 22 (T17L5, 2.4%), 23 (T18L5, 75.8%, T17L6, 11.9%), and 24 (T18L6, 8.8%, T19L5, 1.1%). 

### 3.2. Effect of Vertebral Number on Body Size

In order to evaluate the relationship between the vertebral number and body size and carcass traits, the association analysis were conducted by SNV for the traits, respectively (Table 2). The result showed that the TN, LN and TLN were significantly associated with BL, CHC, and CW. 

### 3.3. Genetic Polymorphism of NR6A1 Gene in Dezhou Donkey

**Figure 1:**
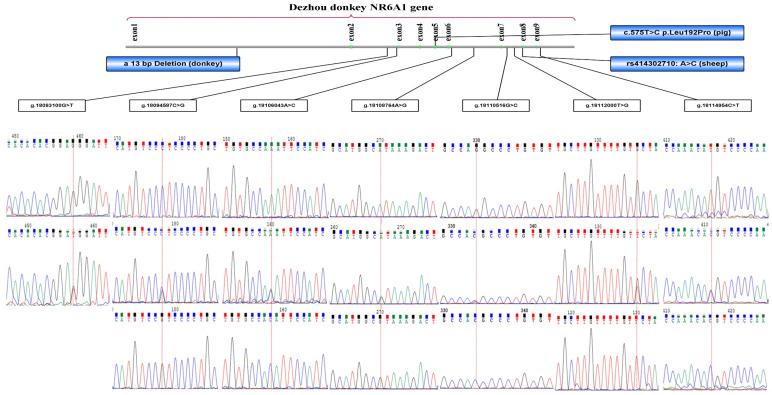
Seven SNVs in the *NR6A1* gene of the Dezhou donkey. Note: The red dotted line indicates the peak map of relevant SNV sites.

The *NR6A1* gene of donkey was located in chromosome 10, consists of nine exons and 10 introns. Seven polymorphism sites including g.18093100G > T, g.18094587C > G, g.18106043A > C, g.18108764A > G, g.18110615G > C, g.18112000T > G and g.18114954C > T were first identified in the introns of the *NR6A1* gene of Dezhou donkey by direct sequencing and comparing these samples with the reference sequence (GenBank accession no. NC_052186.1 Figure 1). The genetic information for the seven loci is shown in Table 3. In the Hardy–Weinberg equilibrium test, six loci were in the Hardy–Weinberg equilibrium (*p* > 0.05). The genetic PIC of *NR6A1* mutation sites ranged from 0.417 to 0.506, which reflects that the genetic diversity of *NR6A1* gene in Dezhou donkeys is moderate.

**Figure 2:**
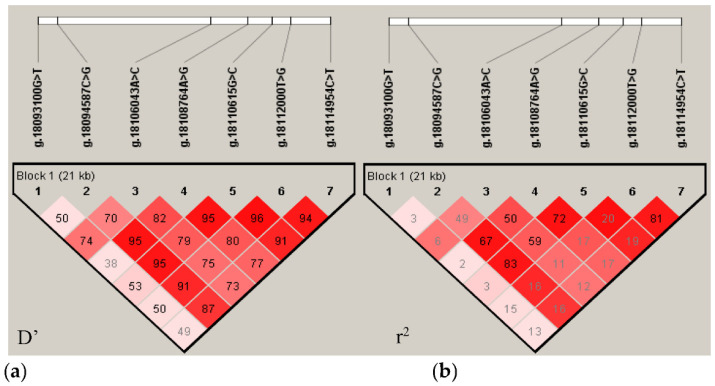
Haplotype constructed by seven SNVs of the *NR6A1* gene. Note: the linkage disequilibrium (LD) plot within 7 SNVs of the *NR6A1* gene was computed by Haploview 4.2 software. The color of each square from light to dark (white to red) indicates the degree of LD from low to high. (**a**) the linkage disequilibrium index of D’, (**b**) the linkage disequilibrium index of r^2^.

In order to reveal the linkage relationship between the seven mutation sites of *NR6A1* gene, D’and r^2^ were calculated to measure the linkage disequilibrium between the seven mutation sites. From Figure 2a (D’) and Figure 2b (r^2^), we can see that there is a strong linkage between g.18094587C > G, g.18106043A > C, g.18108764A > G and g.18110615G > C (r^2^ > 0.33), g.18112000T > G and g.18114954C > T (r^2^ = 0.81).

### 3.4. Association Analysis of Donkey Body Size Traits and Single Loci Polymorphisms 

In order to understand the connection between the SNV locus and body size traits in Dezhou donkey, we analyzed the effects of the seven novel genetic variations on body size traits (Table 4). As such, g.18093100G > T had significantly associated with BH, SW and SLL (the length of single lumbar vertebrae), compared with wild-type homozygotes, mutant homozygotes had significantly lower BH and SLL (*p* < 0.05). g.18094587C > G had significantly associated with BH and CHC (*p* < 0.05). g.18106043A > C and g.18018764A > G associated with CHC significantly (*p* < 0.05). g.18110516G > C and g.18112000T > G associated with BH significantly (*p* < 0.05). g.18114954C > T associated with CW, LN and LTN, heterozygotes have the highest CW, LN and LTN (*p* < 0.05).

### 3.5. Association Analysis of Donkey Body Size Traits and Haplotypes

We constructed 16 haplotypes (*n* ≥ 5) with these seven SNV loci. From Table 5, we can see that Hap1Hap2 is the major haplotype in the experimental population (*n* = 69, 15.2%), the second is Hap1Hap4 and Hap2Hap3 (*n* = 32, 7%), the third is Hap1Hap1 (*n* = 29, 6.4%). Among the 16 haplotypes we constructed, the Hap1Hap8, Hap2Hap5, Hap4Hap4 and Hap4Hap8 had the least number of samples (*n* = 5, 1.1%). A total of 118 samples were not included in the haplotypes we constructed.

We analyzed the association between these 16 haplotypes and thoracolumbar vertebral number as well as body size traits, the results are shown in Table 5. From Table 5, we can see that the significant difference of TL, STL, and STLL exists between these 16 haplotypes (*p* < 0.05), association studies revealed that donkeys with Hap3Hap3 haplotype exhibited significant longer TL, STL and STLL than those with Hap2Hap7, respectively (*p* < 0.05).

## 4. Discussion

Studies in pig, yak and sheep suggested that multi-vertebrae traits contributed to carcass length [12,13,20,28]. Body length of livestock was positively correlated with meat yield and the skin production.

Ample research investigated the range of vertebrae number in different domestic animals. For example, in European ovine breeds like Texel and Scottish Blackface, the range of thoracolumbar number variation was 17~21, and the range of thoracic vertebrae number was 13~14 [29]. The range of thoracolumbar number variation was 18~20 in Chinese Kazakh sheep, the range of thoracic vertebrae number variation was 12~14 [30]. However, the distribution of thoracic and lumbar vertebrae in Dezhou donkey has never been reported. In this study, we precisely collected 455 Dezhou donkeys’ thoracolumbar vertebrae with the slaughter experiment. In Dezhou donkey, T18L6 is the dominant thoracolumbar vertebrae type (75.8%), It has 23 thoracolumbar vertebrae, which was thought to be the ancestral type and all other types may originate from this type. The range of the thoracic vertebrae number was 17~19, the range of lumbar vertebrae number was five to six, and the range of thoracolumbar vertebrae number is 22~24. This was in accordance with the results reported by Jamdar and Ema [17]. Theoretically, there should be individuals with T19L6 thoracolumbar vertebrae type, but we did not observe any. The same situation was also observed in pigs (TN ranged from 14 to 17, LN ranged from five to seven, while TLN ranged from 19 to 23, no T17L7 individual observed [15,31]) and Yak (TN ranged from 14 to 15, LN ranged from four to six, while TLN ranged from 19 to 20, no T15L6 individual was observed [32]). Is this a coincidence? Maybe we need collect more samples. Otherwise, it may be appropriate to study the causes of this situation in the field of spinal dynamics.

It is speculated that the increase in TLN leads to the increase in body length, but this has never been verified statistically and the power of one more thoracic or lumbar vertebrae is still unknown. This study confirmed, at a statistical level, that the increase in the number of thoracolumbar vertebrae of Dezhou donkey is indeed positively correlated with body length and carcass weight. From Table 2, we can see that TN is significantly associated with TL (0.099, *p* < 0.05) and LL (−0.171, *p* < 0.01), with the increase in TN, TL also increasing, while LL decreases significantly, and a similar situation with regards to the opposite correlation in TL and LL occurs when LN changes. Both T18L6 and T19L5 individuals have 24 thoracolumbar vertebrae. With regard to the phenomenon that different types had the same total thoracolumbar vertebrae number, this could be explained by homeotic transformation during vertebrae development [33]. Homeotic transformations do not change the number of vertebrae, but simply their identity (first lumbar to thoracic; last thoracic to lumbar). TLN is significantly associated with BL (0.146, *p* < 0.01) and CW (0.101, *p* < 0.05), one more thoracolumbar vertebrae, a BL increase of about 3 cm, and a CW increase of about 6 kg, roughly. Compared to pigs (one more TLN, BL increase 15 mm [15]) and sheep (one more TLN, TLL increase of about 2.5 cm, CW increase about 1.8 kg [18]), donkeys are especially economic animals; one more thoracolumbar has great economic benefits.

The number of vertebrae in mammals is mainly affected by heredity, for example, the heritability of the number of thoracolumbar vertebrae in pigs is 0.62 [34]. Therefore, the selection of Jack and Jenny with 24 thoracolumbar vertebrae as parents is very important. With the application of artificial insemination technology, most male donkey foals are used for fattening and meat production before they are two years old. Therefore, the development of a simple and efficient molecular detection technology to screen male and female donkeys with multiple thoracolumbar numbers is of great significance to accelerate the breeding of Multi-TLN Dezhou donkeys.

A series of studies have been performed to identify genes regulating vertebrae number variation in livestock. By direct sequencing and association analysis, we found that g.18093100G > T, g.18094587G > T, g.18106043G > T, g.18108764G > T, g.18110615T > G, g.18112000C > T and g.18114954T > G were significantly correlated with body size traits and thoracolumbar vertebrae number in our experimental sample population. It is particularly noteworthy that g.19093100G > T is significantly associated with BH, SW, and SLL. Individuals with TT genotype had significantly lower BH and SLL than wild type and heterozygote (*p* < 0.05), TL, TLL, and CW had the same decreasing trend, but did not reach the significant level. For g.18094587C > G and g.18106043A > C, individuals with mutant genotype had significantly smaller CHC than other genotypes (*p* < 0.05). For g.18108764A > G, individuals with heterozygote had the highest CHC, mutant genotype individuals had significantly smaller CHC, but wild genotype individuals did not differ significantly to others. For g.18112000T > G, individuals with GG and GT genotype had significantly higher CW value than wild type genotype (*p* < 0.05). TN, LN, and TLN did not have such a tendency, but BL, STL, SLL and STLL had the same trend (did not reach a significant level). Therefore, the function of this locus may be to increase carcass length by affecting the length of one vertebra, and then increase carcass weight by increasing carcass length. For g.18114954C > T, individuals with CT genotype had significantly bigger LN and TLN than wild type (*p* < 0.05), but had no significant difference with mutant type. For CW, heterozygous individuals have the highest value, followed by wild-type and mutant homozygous individuals. Statistically, the CW value of mutant homozygous individuals was significantly lower than that of others. It can be seen that different SNVs in the same gene may have contradicting functions in one trait. Moreover, several SNVs have been found associated with CHC, which indicates that these loci in *NR6A1* may have had some functions in the development of ribs.

We constructed 16 haplotypes (n ≥ 5) by seven SNV loci as Haplotype composed of SNVs could provide more accurate information than single loci association analysis for phenotypic traits [14]. We found that individuals with Hap2Hap7 had significantly higher TL, STL and STLL than Hap3Hap3 individuals (*p* < 0.05), while individuals with Hap3Hap3 haplotype had bigger LN and LL than Hap2Hap7 samples (did not reach a significant level). Therefore, we speculate that Hap2Hap7 is the superior haplotype for TL, while Hap3Hap3 is the superior haplotype for the development of lumbar vertebrae.

As a first-class candidate gene, *NR6A1* has been studied in several animals and association with the vertebrae number has been proved. Fang et al. detected a 13 bp Indel in the intron 1 of the Dezhou donkey *NR6A1* gene, and found it was significantly associated with growth traits [35]. Zhang et al. [12] found that there was a significant association between SNV (rs414302710: A > C) in the exon-8 of *NR6A1* gene with the number of lumber vertebrae in sheep (*p* < 0.01). Mikawa et al. [18,19] found that a missense substitution (c.575T > C, p.Pro192Leu) in the *NR6A1* gene was the causative mutation of a quantitative trait locus affecting the number of vertebrae in pigs. Unlike the Asian alleles, all European alleles in that study had the effect of increasing the number of vertebrae by 0.44 to 0.49 and acted additively without dominance. Owing to the lack of pedigree information, it is difficult to calculate the effect of these SNPs on the number of vertebrae between parental generation and offspring. The conclusion in our study is that the function of these variations is complex (positive or negative). Moe et al. [10] found *NR6A1* gene heterozygous genotype (TC) of c.575T > C were observed in the middle-sized indigenous pig (LTN = 21), while homozygous (TT) were fixed in the European commercial breed (Large White, LTN = 22), and homozygous CC were fixed in the Micromini pig (LTN = 19). This loci (c.575T > C) was not found in our study, possibly as the donkey, as an ancient species, did not undergo strong artificial selection. These findings (labeled in Figure 1) prove that the *NR6A1* gene plays an important role in regulating domestic animal vertebrae develop, which also provides support for our results. As far as we know, this is the first study in the word reported that the *NR6A1* gene is related to the body size and thoracolumbar number of donkeys. The TLN, body size or carcass traits are complex quantitative characteristics, which will be influenced by many factors, so the result of this study can only show the effect of SNVs, but it is difficult to quantify the effect.

As a special economic animal, the breeding industry of the Dezhou donkey has gradually become large-scale and standardized in recent years. Dezhou donkeys usually produce only one foal a year, meaning they are less fertile than pigs. Many factors have presented us with great difficulties in trying to establish accurate, systematic and complete Dezhou donkey pedigree records. In conclusion, we still have a lot of work to do to obtain the heritability of the thoracolumbar vertebrae number of Dezhou donkeys for follow-up research.

## 5. Conclusions

In this study, we found that there are five thoracolumbar vertebrae types in Dezhou donkeys (T17L5 2.4%, T18L5 75.8 %, T19L5 1.1%, T17L6 11.9%, and T18L6 8.8%); one more thoracolumbar vertebrate, with a BL increase of about 3 cm, and a CW increase of about 6 kg. Seven SNVs (g.18093100G > T, g.18094587G > T, g.18106043G > T, g.18108764G > T, g.18110615T > G, g.18112000C > T and g.18114954T > G) in this study, and six (besides g.18108764A > G) in Hardy–Weinberg equilibrium state and 0.417 < PIC < 0.506. SNVs and its haplotypes were found to be significantly associated with TLN, body size and carcass traits of the Dezhou donkey (*p* < 0.05), respectively. g.18114954 C > T is significantly associated with LN, TLN, and CW, and individuals with TT genotype had significantly larger LN and TLN than CC genotype (*p* < 0.05). g.18114954C > T site, which can be used as a candidate SNV to study the effects of gene mutations in RNA, protein and regulatory pathways of the development of thoracolumbar vertebrae of Dezhou donkeys. In the haplotypes we constructed, individuals with Hap2Hap7 had significantly larger TL, STL, and STLL than Hap3Hap3.

## Figures and Tables

**Table 1:** Information of amplicon used for PCR.

Primer	Target SNV Site	Primer Sequence (5′–3′)	Tm (°C)	Product Size (bp)	Location
primer 1	g.18093100G > T	F: CACCGTTAGAACGCCACA	60	853	intron 2
		R: GCCTCCACTTACCACCCT			
primer 2	g.18094587C > G	F: GCCCTCACCTTTTGAGGCAA	61	725	intron 2
		R: CTGCAGTTCATTCCCCAGTCA			
primer 3	g.18106043A > C	F: GCTCCATTTCTGCCGGATCT	62	448	intron 6
		R: CCAGGACACATTCCAGAAAATCA			
primer 4	g.18108764A > G	F: TAGGCTAGGGCTCCTTTGCT	63	385	intron 6
		R: GGGCTGAGGTTATGCACAGT			
primer 5	g.18110615G > C	F: GGGGTGCGCAAAAACTTACA	64	643	intron 7
		R: AGTTCACGTGCTCTGCTTCA			
primer 6	g.18112000T > G	F: CACACAGACCACGTGTGAGTA	65	341	intron 7
		R: ACATGTCACAACAGGGGACC			
primer 7	g.18114954C > T	F: CCGATGCATTTCCTTGCAGT	66	596	intron 9
		R: GAGCTGGTAGGCAAGTCTCA			

**Table 2:** The association analysis of TN, LN, and TLN with body size and carcass traits of Dezhou donkeys.

Type	N	BL	CHC	CW	TL	LL	TLL
		(Mean ± SE, cm)	(Mean ± SE, cm)	(Mean ± SE, kg)	(Mean ± SE, cm)	(Mean ± SE, cm)	(Mean ± SE, cm)
17	65	131.33 ± 0.6	141.74 ± 2.29	148.9 ± 1.71	68.86 ± 0.35	26.27 ± 0.24	95.13 ± 0.47
18	385	132.66 ± 0.32	144.66 ± 0.28	152.36 ± 0.86	73.65 ± 0.17	23.78 ± 0.1	97.43 ± 0.23
19	5	132.2 ± 3.85	144.4 ± 2.5	151.5 ± 6.26	75.2 ± 1.59	23.2 ± 0.37	98.4 ± 1.75
Pearson correlation		0.070	0.113 **	0.049	0.099 **	−0.171 ***	0.012
*p* value		0.137	0.016	0.297	0.035	0.000	0.796
5	361	132.28 ± 0.33	144.54 ± 0.29	151.89 ± 0.91	73.53 ± 0.18	23.31 ± 0.08	96.86 ± 0.23
6	94	133.2 ± 0.56	143.1 ± 1.59	151.74 ± 1.41	70.9 ± 0.39	27.23 ± 0.14	98.05 ± 0.48
Pearson correlation		0.060	−0.068	0.040	−0.024	0.312 ***	0.060
*p* value		0.199	0.148	0.390	0.603	0.000	0.200
22	11	128.64 ± 1.44	141.18 ± 1.41	142.3 ± 3.6	68.55 ± 0.79	23.05 ± 0.38	91.59 ± 0.96
23	399	132.32 ± 0.31	144.27 ± 0.45	151.92 ± 0.85	73.03 ± 0.18	23.82 ± 0.1	96.86 ± 0.21
24	45	134.67 ± 0.92	144.73 ± 0.57	153.52 ± 1.99	73.65 ± 0.5	27.09 ± 0.31	100.66 ± 0.66
Pearson correlation		0.146 ***	0.042	0.101 **	0.077 *	0.184 ***	0.084 *
*p* value		0.002	0.376	0.032	0.099	0.000	0.073

Note: *** means *p* < 0.01, Component differences reached a very significant level; ** means 0.01 < *p* < 0.05, Component differences reached significant level; * means 0.05 < *p* < 0.1, There was a significant difference trend between the groups. BL means body length, CHC means chest circumference, CW means carcass weight, TL means the length of thoracic vertebrae, LL means the length of lumbar vertebrae, TLL means the total length of thoracolumbar vertebrae.

**Table 3:** Genotype, allele frequency and genetic parameters of seven SNVs in the *NR6A1* gene in Dezhou donkeys.

Position	Location	Sample	Genotype	Genotype Frequencies	Number	Allele Frequencies		HW		Genetic Parameters			
						Wild	Mutant	Chi-Square	*p* Value	Obs-Het	Pred-Het	PIC	Ne
g.18093100G > T	Intron-2	455	GG	0.64	291	0.7915	0.2085	2.966	0.107	0.303	0.33	0.417	1.5
			GT	0.303	138								
			TT	0.057	26								
g.18094587C > G	Intron-2	455	CC	0.477	217	0.688	0.312	0.118	0.774	0.422	0.429	0.495	1.75
			CG	0.422	192								
			GG	0.101	46								
g.18106043A > C	Intron-6	455	AA	0.455	207	0.686	0.314	2.345	0.166	0.462	0.431	0.478	1.76
			AC	0.462	210								
			CC	0.083	38								
g.18108764A > G	Intron-6	455	AA	0.358	163	0.6175	0.3825	4.465	0.048	0.519	0.472	0.506	1.9
			AG	0.519	236								
			GG	0.123	56								
g.18110516G > C	Intron-7	455	GG	0.455	207	0.6715	0.3285	0.141	0.749	0.433	0.441	0.505	1.79
			GC	0.433	197								
			CC	0.112	51								
g.18112000T > G	Intron-7	455	TT	0.101	46	0.6955	0.3045	0.682	0.444	0.407	0.423	0.494	1.74
			GT	0.407	185								
			GG	0.492	224								
g.18114954C > T	downstream	455	CC	0.444	202	0.3245	0.6755	1.603	0.264	0.464	0.438	0.488	1.78
			TC	0.463	211								
			TT	0.093	42								

**Table 4:** Multiple comparisons between genotypes of seven SNVs and body size traits, slaughter traits and thoracolumbar character in Dezhou donkeys.

SNV Site	Genetype/ Number	BH	BL	CHC	SW	CW	TN	TL	STL	LN	LL	SLL	TLN	LTL	STLL
g.18093100G > T	GG/291	134.95 ± 0.31 ^a^	132.62 ± 0.37	144.44 ± 0.59	24.39 ± 0.23 ^b^	151.89 ± 0.98	17.87 ± 0.02	73.02 ± 0.22	4.09 ± 0.01	5.20 ± 0.02	24.14 ± 0.13	4.64 ± 0.02 ^a^	23.07 ± 0.02	97.17 ± 0.28	4.21 ± 0.01
	GT/138	134.65 ± 0.41 ^a^	132.65 ± 0.50	143.96 ± 0.44	25.51 ± 0.44 ^a^	152.88 ± 1.40	17.88 ± 0.03	73.09 ± 0.29	4.09 ± 0.02	5.22 ± 0.04	24.25 ± 0.17	4.65 ± 0.02 ^a^	23.09 ± 0.03	97.36 ± 0.35	4.21 ± 0.01
	TT/26	131.77 ± 1.03 ^b^	129.77 ± 1.15	143.52 ± 0.99	23.82 ± 0.90 ^ab^	146.10 ± 2.51	17.85 ± 0.07	71.90 ± 0.67	4.03 ± 0.03	5.19 ± 0.08	23.38 ± 0.45	4.49 ± 0.05 ^b^	23.04 ± 0.07	95.10 ± 0.67	4.13 ± 0.03
	*p* value	0.011	0.072	0.789	0.028	0.171	0.915	0.299	0.285	0.925	0.185	0.032	0.663	0.065	0.079
g.18094587C > G	CC/217	134.01 ± 0.35 ^b^	132.06 ± 0.42	144.26 ± 0.35 ^a^	24.81 ± 0.32	151.45 ± 1.12	17.86 ± 0.02	72.82 ± 0.25	4.08 ± 0.01	5.19 ± 0.03	23.90 ± 0.13	4.62 ± 0.02	23.05 ± 0.02	96.70 ± 0.29	4.20 ± 0.01
	CG/192	135.40 ± 0.36 ^a^	133.05 ± 0.43	144.89 ± 0.40 ^a^	24.55 ± 0.31	152.78 ± 1.22	17.87 ± 0.03	73.08 ± 0.25	4.09 ± 0.01	5.23 ± 0.03	24.40 ± 0.17	4.66 ± 0.02	23.10 ± 0.03	97.51 ± 0.31	4.22 ± 0.01
	GG/46	134.85 ± 0.81 ^ab^	131.96 ± 1.03	141.41 ± 3.23 ^b^	24.75 ± 0.52	149.89 ± 2.23	17.91 ± 0.06	73.29 ± 0.64	4.09 ± 0.03	5.20 ± 0.06	24.07 ± 0.38	4.62 ± 0.05	23.11 ± 0.06	97.38 ± 0.85	4.21 ± 0.03
	*p* value	0.025	0.222	0.048	0.836	0.497	0.647	0.64	0.701	0.595	0.071	0.243	0.233	0.179	0.414
g.18106043A > C	AA/207	134.15 ± 0.35	132.43 ± 0.43	144.33 ± 0.36 ^a^	24.83 ± 0.32	152.38 ± 1.15	17.84 ± 0.03	72.69 ± 0.25	4.07 ± 0.01	5.24 ± 0.03	24.16 ± 0.15	4.62 ± 0.02	23.08 ± 0.02	96.81 ± 0.29	4.20 ± 0.01
	AC/210	135.31 ± 0.37	132.65 ± 0.44	144.95 ± 0.39 ^a^	24.67 ± 0.31	151.48 ± 1.19	17.90 ± 0.02	73.33 ± 0.26	4.10 ± 0.01	5.16 ± 0.03	24.06 ± 0.15	4.66 ± 0.02	23.06 ± 0.02	97.42 ± 0.32	4.22 ± 0.01
	CC/38	134.05 ± 0.74	131.71 ± 0.93	139.87 ± 3.83 ^b^	24.12 ± 0.38	151.21 ± 1.93	17.87 ± 0.07	72.55 ± 0.55	4.06 ± 0.03	5.26 ± 0.07	24.38 ± 0.44	4.62 ± 0.04	23.13 ± 0.08	96.98 ± 0.81	4.19 ± 0.03
	*p* value	0.052	0.687	0.003	0.653	0.829	0.208	0.155	0.334	0.089	0.705	0.264	0.511	0.377	0.231
g.18108764A > G	AA/163	133.91 ± 0.39	132.12 ± 0.47	143.74 ± 0.4 ^ab^	24.78 ± 0.39	150.93 ± 1.28	17.82 ± 0.03	72.45 ± 0.29	4.07 ± 0.01	5.25 ± 0.03	24.07 ± 0.17	4.60 ± 0.02	23.06 ± 0.02	96.48 ± 0.34	4.18 ± 0.01
	AG/236	135.16 ± 0.34	132.79 ± 0.40	145.11 ± 0.36 ^a^	24.62 ± 0.27	152.59 ± 1.10	17.90 ± 0.02	73.25 ± 0.22	4.09 ± 0.01	5.19 ± 0.03	24.17 ± 0.14	4.66 ± 0.02	23.08 ± 0.02	97.44 ± 0.28	4.22 ± 0.01
	GG/56	134.91 ± 0.71	132.13 ± 0.89	142.04 ± 2.67 ^b^	24.78 ± 0.46	151.40 ± 2.04	17.89 ± 0.06	73.33 ± 0.58	4.10 ± 0.03	5.18 ± 0.05	24.13 ± 0.33	4.65 ± 0.04	23.07 ± 0.06	97.51 ± 0.76	4.23 ± 0.03
	*p* value	0.056	0.517	0.036	0.926	0.605	0.079	0.071	0.247	0.31	0.895	0.094	0.798	0.088	0.117
g.18110615G > C	GG/207	134.00 ± 0.36 ^b^	132.17 ± 0.43	144.16 ± 0.36	24.90 ± 0.33	151.41 ± 1.16	17.86 ± 0.02	72.75 ± 0.25	4.07 ± 0.01	5.20 ± 0.03	23.94 ± 0.14	4.61 ± 0.02	23.06 ± 0.02	96.67 ± 0.30	4.19 ± 0.01
	GC/197	135.41 ± 0.36 ^a^	132.92 ± 0.43	144.97 ± 0.39	24.51 ± 0.30	152.57 ± 1.20	17.88 ± 0.03	73.18 ± 0.25	4.09 ± 0.01	5.21 ± 0.03	24.34 ± 0.16	4.67 ± 0.02	23.09 ± 0.03	97.54 ± 0.32	4.22 ± 0.01
	CC/51	134.59 ± 0.74 ^ab^	131.94 ± 0.94	141.77 ± 2.92	24.63 ± 0.49	150.94 ± 2.10	17.88 ± 0.06	73.10 ± 0.59	4.09 ± 0.03	5.20 ± 0.06	24.07 ± 0.37	4.62 ± 0.05	23.08 ± 0.06	97.22 ± 0.79	4.21 ± 0.03
	*p* value	0.023	0.39	0.06	0.67	0.713	0.788	0.483	0.511	0.95	0.189	0.131	0.619	0.152	0.248
g.18112000T > G	TT/46	133.11 ± 0.79 ^b^	131.67 ± 0.85	142.7 ± 0.77	24.19 ± 0.83	146.05 ± 2.52 ^b^	17.85 ± 0.05	72.5 ± 0.53	4.06 ± 0.03	5.26 ± 0.07	24.05 ± 0.31	4.59 ± 0.04	23.11 ± 0.05	96.55 ± 0.61	4.18 ± 0.03
	GT/185	134.54 ± 0.40 ^ab^	132.56 ± 0.48	144.51 ± 0.39	25.15 ± 0.34	153.17 ± 1.31 ^a^	17.89 ± 0.03	73.06 ± 0.27	4.08 ± 0.01	5.16 ± 0.03	23.92 ± 0.15	4.63 ± 0.02	23.05 ± 0.02	96.99 ± 0.32	4.21 ± 0.01
	GG/224	135.12 ± 0.33 ^a^	132.55 ± 0.40	144.34 ± 0.73	24.43 ± 0.26	151.99 ± 1.02 ^a^	17.86 ± 0.03	73.00 ± 0.24	4.09 ± 0.01	5.23 ± 0.03	24.32 ± 0.16	4.65 ± 0.02	23.09 ± 0.03	97.32 ± 0.31	4.21 ± 0.01
	*p* value	0.050	0.657	0.431	0.176	0.033	0.674	0.641	0.580	0.140	0.171	0.397	0.383	0.518	0.449
g.18114954C > T	CC/202	134.87 ± 0.34	132.16 ± 0.41	144.38 ± 0.81	24.43 ± 0.28	151.74 ± 1.07 ^a^	17.87 ± 0.03	72.93 ± 0.25	4.08 ± 0.01	5.15 ± 0.03 ^b^	23.94 ± 0.14	4.65 ± 0.02	23.01 ± 0.02 ^b^	96.87 ± 0.30	4.21 ± 0.01
	TC/211	134.75 ± 0.37	132.91 ± 0.45	144.41 ± 0.36	25.05 ± 0.31	153.3 ± 1.19 ^a^	17.88 ± 0.02	73.16 ± 0.26	4.09 ± 0.01	5.26 ± 0.03 ^a^	24.37 ± 0.16	4.63 ± 0.02	23.14 ± 0.03 ^a^	97.52 ± 0.32	4.21 ± 0.01
	TT/42	133.40 ± 0.84	131.76 ± 0.88	142.73 ± 0.82	24.23 ± 0.90	145.25 ± 2.70 ^b^	17.83 ± 0.06	72.33 ± 0.56	4.05 ± 0.03	5.21 ± 0.06 ^ab^	23.91 ± 0.31	4.60 ± 0.04	23.05 ± 0.03 ^ab^	96.24 ± 0.65	4.18 ± 0.03
	*p* value	0.240	0.348	0.489	0.277	0.016	0.783	0.395	0.411	0.019	0.113	0.599	0.001	0.146	0.461

Note: Phenotypic values are shown in mean ± standard deviation. Values with the same superscript or without superscript in the same column mean no significant difference. Values with different superscripts in the same line are significantly different (*p* < 0.05).

**Table 5:** Association analysis of Haplotypes of *NR6A1* gene and body size traits, slaughter traits and thoracolumbar character in Dezhou donkeys.

	n	BH	BL	CHC	SW	CW	TN	TL	STL	LN	LL	SLL	TLN	TLL	STLL
Hap1Hap2	69	135.25 ± 0.57	132.44 ± 0.70	145.49 ± 0.71	24.11 ± 0.58	151.90 ± 2.13	17.87 ± 0.04	73.12 ± 0.35 ^AB^	4.09 ± 0.02 ^ab^	5.14 ± 0.04	24.08 ± 0.28	4.68 ± 0.05	23.01 ± 0.03	97.20 ± 0.50	4.22 ± 0.02 ^ab^
Hap1Hap4	32	133.38 ± 0.91	132.06 ± 0.91	143.8 ± 0.94	25.69 ± 0.84	154.64 ± 2.87	17.69 ± 0.08	71.67 ± 0.57 ^AB^	4.05 ± 0.03 ^ab^	5.34 ± 0.09	24.36 ± 0.43	4.56 ± 0.03	23.03 ± 0.07	96.03 ± 0.65	4.17 ± 0.02 ^ab^
Hap2Hap3	32	136.28 ± 0.97	134.00 ± 0.98	145.47 ± 0.97	25.57 ± 0.61	154.60 ± 2.98	17.84 ± 0.08	73.62 ± 0.70 ^AB^	4.13 ± 0.03 ^ab^	5.19 ± 0.07	24.41 ± 0.34	4.71 ± 0.05	23.03 ± 0.03	98.16 ± 0.69	4.26 ± 0.03 ^ab^
Hap1Hap1	29	134.69 ± 0.90	132.24 ± 0.95	146.28 ± 0.87	25.01 ± 0.90	154.94 ± 2.27	17.76 ± 0.08	71.68 ± 0.67 ^AB^	4.04 ± 0.03 ^ab^	5.24 ± 0.08	24.30 ± 0.36	4.64 ± 0.04	23.00 ± 0.00	95.98 ± 0.67	4.17 ± 0.03 ^ab^
Hap1Hap3	27	134.07 ± 1.03	132.54 ± 1.63	143.33 ± 1.16	24.34 ± 0.91	151.67 ± 4.26	17.96 ± 0.04	73.35 ± 0.77 ^AB^	4.08 ± 0.04 ^ab^	5.11 ± 0.06	23.4 ± 0.35	4.58 ± 0.05	23.07 ± 0.05	96.75 ± 0.95	4.19 ± 0.04 ^ab^
Hap2Hap2	25	134.16 ± 1.02	131.32 ± 1.11	137.54 ± 5.77	23.65 ± 0.45	147.96 ± 2.08	17.92 ± 0.08	72.26 ± 0.71 ^AB^	4.03 ± 0.04 ^ab^	5.20 ± 0.08	23.85 ± 0.47	4.58 ± 0.05	23.12 ± 0.09	96.15 ± 0.95	4.16 ± 0.04 ^ab^
Hap2Hap4	25	135.60 ± 1.15	133.16 ± 1.34	144.2 ± 1.17	25.28 ± 1.23	152.37 ± 3.67	17.92 ± 0.06	73.22 ± 0.77 ^AB^	4.09 ± 0.04 ^ab^	5.16 ± 0.07	24.45 ± 0.5	4.72 ± 0.06	23.08 ± 0.06	97.77 ± 1.03	4.23 ± 0.04 ^ab^
Hap3Hap4	13	136.42 ± 1.39	134.92 ± 1.39	144.04 ± 1.57	27.72 ± 2.51	154.05 ± 5.20	17.77 ± 0.12	75.18 ± 1.14 ^AB^	4.22 ± 0.05 ^a^	5.23 ± 0.12	24.64 ± 0.34	4.77 ± 0.10	23.00 ± 0.00	99.82 ± 1.21	4.34 ± 0.05 ^ab^
Hap2Hap7	9	137.33 ± 2.49	135.00 ± 3.71	148.67 ± 2.43	25.89 ± 1.18	153.78 ± 8.40	18.00 ± 0.17	76.11 ± 1.87 ^A^	4.23 ± 0.10 ^a^	5.00 ± 0.00	23.61 ± 0.87	4.72 ± 0.17	23.00 ± 0.17	99.72 ± 2.53	4.34 ± 0.11 ^a^
Hap1Hap5	8	133.75 ± 1.25	130.25 ± 1.72	144.5 ± 1.13	24.26 ± 0.97	151.75 ± 3.51	18.00 ± 0.00	73.00 ± 1.07 ^AB^	4.06 ± 0.06 ^ab^	5.00 ± 0.00	23.75 ± 0.49	4.75 ± 0.10	23.00 ± 0.00	96.75 ± 1.44	4.21 ± 0.06 ^ab^
Hap1Hap6	8	134.31 ± 1.33	131.5 ± 1.88	144.94 ± 2.35	24.04 ± 0.84	154.06 ± 7.23	17.75 ± 0.16	71.19 ± 1.09 ^B^	4.01 ± 0.05 ^ab^	5.38 ± 0.18	24.56 ± 0.43	4.59 ± 0.09	23.13 ± 0.13	95.75 ± 0.88	4.14 ± 0.05 ^ab^
Hap3Hap3	8	132.56 ± 1.50	131.00 ± 1.66	141.81 ± 1.31	22.74 ± 0.81	141.31 ± 5.19	17.75 ± 0.16	69.88 ± 0.99 ^B^	3.94 ± 0.05 ^b^	5.38 ± 0.18	24.06 ± 0.91	4.48 ± 0.08	23.13 ± 0.13	93.94 ± 1.21	4.06 ± 0.05 ^b^
Hap1Hap8	5	136.00 ± 2.86	131.80 ± 3.20	147.8 ± 1.93	24.46 ± 1.40	159.7 ± 5.85	17.80 ± 0.20	74.00 ± 2.28 ^AB^	4.15 ± 0.09 ^ab^	5.00 ± 0.00	23.18 ± 0.34	4.64 ± 0.07	22.80 ± 0.20	97.18 ± 2.59	4.26 ± 0.08 ^ab^
Hap2Hap5	5	135.20 ± 2.13	134.80 ± 1.59	141.60 ± 3.08	23.50 ± 0.99	151.30 ± 7.65	18.20 ± 0.20	74.80 ± 0.41 ^AB^	4.11 ± 0.03 ^ab^	5.00 ± 0.00	22.80 ± 0.37	4.56 ± 0.07	23.20 ± 0.20	97.60 ± 0.53	4.21 ± 0.04 ^ab^
Hap4Hap4	5	131.60 ± 2.75	130.80 ± 2.56	140.20 ± 2.07	21.62 ± 1.46	137.80 ± 6.50	17.80 ± 0.20	71.70 ± 1.95 ^AB^	4.03 ± 0.07 ^ab^	5.20 ± 0.20	23.80 ± 1.15	4.58 ± 0.14	23.00 ± 0.00	95.50 ± 1.78	4.15 ± 0.08 ^ab^
Hap4Hap8	5	132.80 ± 2.08	128.60 ± 4.21	143.10 ± 2.04	24.28 ± 1.18	144.80 ± 7.28	18.20 ± 0.20	74.10 ± 1.95 ^AB^	4.08 ± 0.14 ^ab^	5.00 ± 0.00	23.20 ± 0.58	4.64 ± 0.12	23.20 ± 0.20	97.30 ± 2.45	4.20 ± 0.13 ^ab^
*p*-value		0.419	0.702	0.230	0.398	0.593	0.078	0.006	0.020	0.204	0.748	0.412	0.689	0.133	0.040

Note: Phenotypic values are shown in mean ± standard deviation. Values with the same superscript or without superscript in the same column mean no significant difference. Values with different superscripts in the same line are significantly different (*p* < 0.05).

## Data Availability

The data presented in this study are available on request from the corresponding author.

## Supplementary Materials

The following supporting information can be downloaded at: https://www.mdpi.com/article/10.3390/ani12111349/s1, Table S1. Information of amplicon used for amplifying the total region on NR6A1 gene.

## References

[1] Polidori P., Pucciarelli S., Ariani A., Polzonetti V., Vincenzetti S. (2015). A comparison of the carcass and meat quality of Martina Franca donkey foals aged 8 or 12 months. Meat Sci..

[2] Wang C., Li H., Guo Y., Huang J., Sun Y., Min J., Wang J., Fang X., Zhao Z., Wang S. (2020). Donkey genomes provide new insights into domestication and selection for coat color. Nat. Commun..

[3] Denham T., Iriarte J., Vrydaghs L. (2009). Rethinking Agriculture: Archaeological and Ethnoarchaeological Perspectives. J. Anthropol. Res..

[4] Wellik D.M. (2007). Hox patterning of the vertebrate axial skeleton. Dev. Dyn..

[5] Kaiser J.T., Reddy V., Lugo-Pico J.G. (2022). Anatomy, Head and Neck, Cervical Vertebrae. StatPearls.

[6] Galis F. (1999). Why do almost all mammals have seven cervical vertebrae? Developmental constraints, Hox genes, and cancer. J. Exp. Zool..

[7] Faraz A., Tirink C., Eyduran E., Waheed A., Tauqir N.A., Nabeel M.S., Tariq M.M. (2021). Prediction of live body weight based on body measurements in Thalli sheep under tropical conditions of Pakistan using cart and mars. Trop. Anim. Health Prod..

[8] Fredeen H.T., Newman J.A. (1962). Rib and Vertebral Numbers in Swine. Can. J. Anim. Sci..

[9] Liu Q., Yue J., Niu N., Liu X., Yan H., Zhao F., Hou X., Gao H., Shi L., Wang L. (2020). Genome-Wide Association Analysis Identified BMPR1A as a Novel Candidate Gene Affecting the Number of Thoracic Vertebrae in a Large White x Minzhu Intercross Pig Population. Animals.

[10] Ijiri M., Lai Y.C., Kawaguchi H., Fujimoto Y., Miura N., Matsuo T., Tanimoto A. (2021). NR6A1 Allelic Frequencies as an Index for both Miniaturizing and Increasing Pig Body Size. In Vivo.

[11] Li C., Li M., Li X., Ni W., Xu Y., Yao R., Wei B., Zhang M., Li H., Zhao Y. (2019). Whole-Genome Resequencing Reveals Loci Associated With Thoracic Vertebrae Number in Sheep. Front. Genet..

[12] Zhang X., Li C., Li X., Liu Z., Ni W., Cao Y., Yao Y., Islamov E., Wei J., Hou X. (2019). Association analysis of polymorphism in the NR6A1 gene with the lumbar vertebrae number traits in sheep. Genes Genom..

[13] Li C., Zhang X., Cao Y., Wei J., You S., Jiang Y., Cai K., Wumaier W., Guo D., Qi J. (2017). Multi-vertebrae variation potentially contribute to carcass length and weight of Kazakh sheep. Small Rumin. Res..

[14] Raza S.H.A., Khan R., Gui L., Schreurs N.M., Wang X., Mei C., Yang X., Gong C., Zan L. (2020). Bioinformatics analysis and genetic polymorphisms in genomic region of the bovine SH2B2 gene and their associations with molecular breeding for body size traits in qinchuan beef cattle. Biosci. Rep..

[15] King J.W.B., Roberts R.C. (2010). Carcass length in the bacon pig; its association with vertebrae numbers and prediction from radiographs of the young pig. Anim. Prod..

[16] Stecher R.M. (1962). Activity of Small Mammals as Recorded By a Photographic Device. J. Mammal..

[17] Jamdar M.N., Ema A.N. (1982). A Note on the Vertebral Formula of the Donkey. Br. Vet. J..

[18] Mikawa S., Hayashi T., Nii M., Shimanuki S., Morozumi T., Awata T. (2005). Two quantitative trait loci on Sus scrofa chromosomes 1 and 7 affecting the number of vertebrae. J. Anim. Sci..

[19] Mikawa S., Morozumi T., Shimanuki S., Hayashi T., Uenishi H., Domukai M., Okumura N., Awata T. (2007). Fine mapping of a swine quantitative trait locus for number of vertebrae and analysis of an orphan nuclear receptor, germ cell nuclear factor (NR6A1). Genome Res..

[20] Mikawa S., Sato S., Nii M., Morozumi T., Yoshioka G., Imaeda N., Yamaguchi T., Hayashi T., Awata T. (2011). Identification of a second gene associated with variation in vertebral number in domestic pigs. BMC Genet..

[21] Yang G., Ren J., Zhang Z., Huang L. (2009). Genetic evidence for the introgression of Western NR6A1 haplotype into Chinese Licha breed associated with increased vertebral number. Anim. Genet..

[22] Zhang Y., Wang M., Yuan J., Zhou X., Xu S., Liu B. (2018). Association of polymorphisms in NR6A1, PLAG1 and VRTN with the number of vertebrae in Chinese Tongcheng x Large White crossbred pigs. Anim. Genet..

[23] Maeda S., Koya D., Araki S.I., Babazono T., Umezono T., Toyoda M., Kawai K., Imanishi M., Uzu T., Suzuki D. (2011). Association between single nucleotide polymorphisms within genes encoding sirtuin families and diabetic nephropathy in Japanese subjects with type 2 diabetes. Clin. Exp. Nephrol..

[24] Zhang Z., Zhan Y., Han Y., Liu Z., Wang Y., Wang C. (2021). Estimation of Liveweight from Body Measurements through Best Fitted Regression Model in Dezhou Donkey Breed. J. Equine Vet. Sci..

[25] Nei M., Roychoudhury A.K. (1974). Sampling variances of heterozygosity and genetic distance. Genetics.

[26] Shi Y.Y., He L. (2005). SHEsis, a powerful software platform for analyses of linkage disequilibrium, haplotype construction, and genetic association at polymorphism loci. Cell Res..

[27] Barrett J.C., Fry B., Maller J., Daly M.J. (2005). Haploview: Analysis and visualization of LD and haplotype maps. Bioinformatics.

[28] Wang Y., Cai H., Luo X., Ai Y., Jiang M., Wen Y. (2020). Insight into unique somitogenesis of yak (*Bos grunniens*) with one additional thoracic vertebra. BMC Genom..

[29] Donaldson C.L., Lambe N.R., Maltin C.A., Knott S., Bunger L. (2013). Between- and within-breed variations of spine characteristics in sheep. J. Anim. Sci..

[30] Zhang Z., Sun Y., Du W., He S., Liu M., Tian C. (2017). Effects of vertebral number variations on carcass traits and genotyping of Vertnin candidate gene in Kazakh sheep. Asian-Australas J. Anim. Sci..

[31] Stecher R.M. (1962). Anatomical Variations of The Spine in The Horse. J. Mammal..

[32] Yang J., Wen Y., Feng Z., Ke m., Gao X., An D. (2015). Variation of Thoracic and Lumbar Vertebrae of Jinchuan Yak and its Correlation with Meat Production Performance. J. Livest. Ecol..

[33] van den Akker E., Forlani S., Chawengsaksophak K., de Graaff W., Beck F., Meyer B.I., Deschamps J. (2002). Cdx1 and Cdx2 have overlapping functions in anteroposterior patterning and posterior axis elongation. Development.

[34] van Son M., Lopes M.S., Martell H.J., Derks M.F.L., Gangsei L.E., Kongsro J., Wass M.N., Grindflek E.H., Harlizius B. (2019). A QTL for Number of Teats Shows Breed Specific Effects on Number of Vertebrae in Pigs: Bridging the Gap Between Molecular and Quantitative Genetics. Front. Genet..

[35] Fang X., Lai Z., Liu J., Zhang C., Li S., Wu F., Zhou Z., Lei C., Dang R. (2019). A Novel 13 bp Deletion within the NR6A1 Gene Is Significantly Associated with Growth Traits in Donkeys. Animals.

